# Identification of Succinate Dehydrogenase Gene Variant Carriers by Blood Biomarkers

**DOI:** 10.1210/jendso/bvae142

**Published:** 2024-08-04

**Authors:** Marcel Gebhardt, Carola Kunath, Dennis Fröbel, Alexander M Funk, Mirko Peitzsch, Svenja Nölting, Timo Deutschbein, Andrzej Januszewicz, Henri J L M Timmers, Mercedes Robledo, Arne Jahn, Georgiana Constantinescu, Graeme Eisenhofer, Christina Pamporaki, Susan Richter

**Affiliations:** Institute for Clinical Chemistry and Laboratory Medicine, Faculty of Medicine, University Hospital Carl Gustav Carus, Technische Universität Dresden, 01307 Dresden, Germany; Department of Internal Medicine III, University Hospital Carl Gustav Carus, Technische Universität Dresden, 01307 Dresden, Germany; Institute for Clinical Chemistry and Laboratory Medicine, Faculty of Medicine, University Hospital Carl Gustav Carus, Technische Universität Dresden, 01307 Dresden, Germany; Institute for Clinical Chemistry and Laboratory Medicine, Faculty of Medicine, University Hospital Carl Gustav Carus, Technische Universität Dresden, 01307 Dresden, Germany; Institute for Clinical Chemistry and Laboratory Medicine, Faculty of Medicine, University Hospital Carl Gustav Carus, Technische Universität Dresden, 01307 Dresden, Germany; Medizinische Klinik and Poliklinik IV, Ludwig-Maximilians-Universität München, D-80336 Munich, Germany; Department for Endocrinology, Diabetology and Clinical Nutrition, UniversitätsSpital Zürich, 8091 Zurich, Switzerland; Department of Internal Medicine I, Division of Endocrinology and Diabetes, University Hospital, University of Würzburg, 97080 Würzburg, Germany; Medicover Oldenburg MVZ, 26122 Oldenburg, Germany; Department of Hypertension, National Institute of Cardiology, 04-628 Warsaw, Poland; Department of Internal Medicine, Radboud University Medical Centre, 6265 Nijmegen, The Netherlands; Hereditary Endocrine Cancer Group, CNIO, 28029 Madrid, Spain; Centro de Investigación Biomédica en Red de Enfermedades Raras (CIBERER), 28029 Madrid, Spain; Institute for Clinical Genetics, University Hospital Carl Gustav Carus at TUD Dresden University of Technology and Faculty of Medicine of TUD Dresden University of Technology, 01307 Dresden, Germany; ERN GENTURIS, Hereditary Cancer Syndrome Center Dresden, 01307 Dresden, Germany; National Center for Tumor Diseases (NCT), NCT/UCC Dresden, a partnership between German Cancer Research Center (DKFZ), Faculty of Medicine and University Hospital Carl Gustav Carus, TUD Dresden University of Technology and Helmholtz-Zentrum Dresden-Rossendorf (HZDR), 01307 Dresden, Germany; German Cancer Consortium (DKTK), 01307 Dresden, Germany; German Cancer Research Center (DKFZ), 69120 Heidelberg, Germany; Max Planck Institute of Molecular Cell Biology and Genetics, 01307 Dresden, Germany; Department of Internal Medicine III, University Hospital Carl Gustav Carus, Technische Universität Dresden, 01307 Dresden, Germany; Department of Internal Medicine III, University Hospital Carl Gustav Carus, Technische Universität Dresden, 01307 Dresden, Germany; Department of Internal Medicine III, University Hospital Carl Gustav Carus, Technische Universität Dresden, 01307 Dresden, Germany; Institute for Clinical Chemistry and Laboratory Medicine, Faculty of Medicine, University Hospital Carl Gustav Carus, Technische Universität Dresden, 01307 Dresden, Germany

**Keywords:** succinate dehydrogenase gene variants, blood plasma, urine, erythrocytes, PBMC, metabolism

## Abstract

**Background:**

Carriers of germline pathogenic variants (PVs) in succinate dehydrogenase genes (*SDHx*) are at risk of developing tumors, including paragangliomas, gastrointestinal stromal tumors, and renal cell carcinomas. Early tumor detection is paramount for improved clinical outcome. Blood-based biomarkers could aid in identifying individuals with PVs early and provide functional evidence in patients with variants of unknown significance.

**Methods:**

Blood plasma, urine, peripheral blood mononuclear cells, and erythrocytes from patients with and without *SDHx* PVs were investigated for central carbon metabolites. These were measured by liquid chromatography–tandem mass spectrometry and nuclear magnetic resonance spectroscopy and included among others, succinate, fumarate, α-ketoglutarate, and lactate.

**Results:**

Plasma succinate to fumarate ratios effectively distinguished tumor-bearing and asymptomatic patients with and without *SDHx* PV with promising diagnostic performance (areas under the receiver operating characteristic curve 0.86-0.95), although higher levels were noted in individuals with *SDHB* PV. Metabolites in urine and in peripheral blood mononuclear cell extracts were largely similar between groups. Erythrocytes showed strong metabolic alterations in patients with *SDHx* PV compared to controls, with 8 of 13 low-molecular organic acids being significantly different (*P* < .05). The lactate-α-ketoglutarate-ratio of erythrocytes identified individuals with *SDHx* PV equally well as plasma, with a sensitivity and specificity of 92% (AUC 0.97).

**Conclusion:**

Blood biomarkers have been underutilized for identifying carriers of *SDHx* PV or to validate variants of unknown significance. Our findings advocate for further investigation into a combined approach involving plasma and erythrocytes for future diagnostic strategies.

Succinate dehydrogenase (SDH) is an enzyme complex located at the inner mitochondrial membrane, where it participates in metabolism of the Krebs cycle and also facilitates electron transfer within the respiratory chain as complex II. SDH comprises 4 subunits: SDHA is catalytically active and converts succinate to fumarate. SDHB contains Fe-S clusters for electron transfer, and SDHC and SDHD are membrane anchors. Heterozygous loss-of-function germline variants in succinate dehydrogenase genes (*SDHA, SDHB, SDHC, SDHD*, and assembly factor *SDHAF2*) are responsible for a number of tumor diseases, foremostly paragangliomas of the sympathetic or parasympathetic nervous system and pheochromocytomas (together referred to as PPGL). Carriers of germline pathogenic variants (PVs) in *SDHx* can also develop gastrointestinal stromal tumors, renal cell carcinomas, and rarely, pituitary adenomas or thyroid cancer [1]. Due to this risk profile, lifelong tumor screening is recommended for *SDHx* PV carriers [2]. Potential benefits of such a screening approach are evident from the literature. Early surveillance resulted in reduced metastatic spread in patients with *SDHx* PVs and in increased metastasis-free and overall survival in patients with PVs in *SDHB* [3, 4].

The basis of any surveillance program requires correct identification of individuals at risk. Low penetrance of disease can hinder the recognition of family transmission leading to *SDHx* PV detection earliest at the stage of tumor presentation [5-8]. Furthermore, variant classification is especially difficult for missense variants in *SDHx* genes. Currently 3334 variants of uncertain significance (VUS) or with conflicting interpretations are listed for *SDHx* genes and the assembly factor *SDHAF2* in the ClinVar database (accessed March 8, 2024).

Several methods have been described to identify patients with tumors due to *SDHx* PVs, including ex vivo measurements of the succinate to fumarate ratio (SFR) in tumor tissue or the SDHB protein by immunohistochemistry as well as in situ detection of succinate by proton magnetic resonance spectroscopy [9]. As complete loss of SDH activity results in a blockade of the Krebs cycle with strong accumulation of the precursor succinate and a decrease of the product fumarate, elevated succinate or SFR levels in the tumor are important diagnostic parameters that can aid variant classification [10, 11]. More recently, it was demonstrated that serum succinate levels are elevated in patients with *SDHB* PVs and PPGL as well as in asymptomatic carriers [12, 13]. Our study aims to validate and extend these results by not only looking at the liquid parts of blood, that is, the serum or plasma, but also at cellular components, such as peripheral mononuclear cells (PBMCs) and erythrocytes. Therefore, we investigated these parameters in patients with a history of PPGL before and/or after tumor removal.

## Methods

### Study Participants, Clinical Data, and Sample Collection

**Figure 1.**
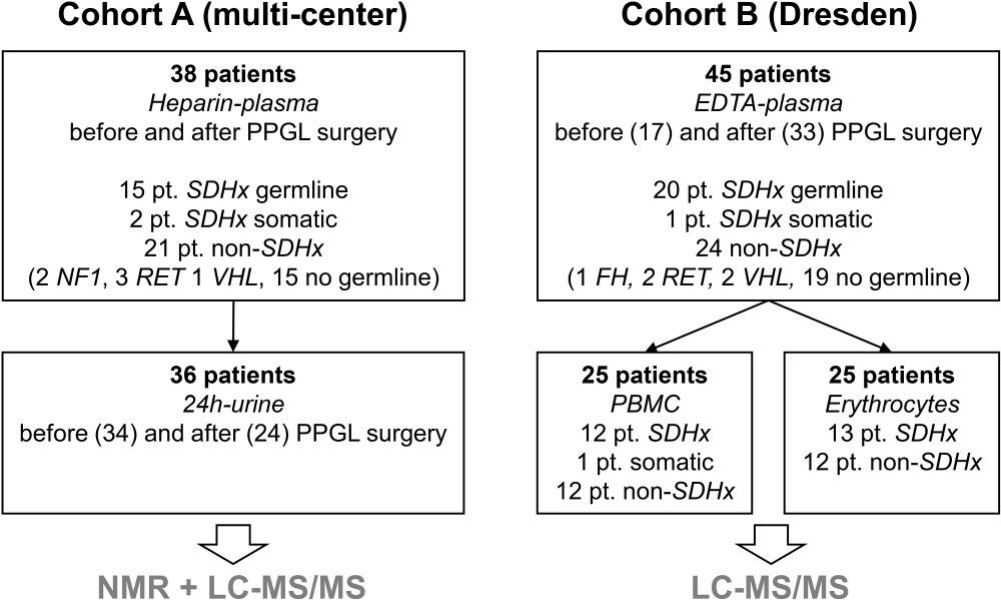
Overview of patient (pt.) cohorts and available material for LC-MS/MS and NMR. A detailed breakdown of patients can be found in Supplementary Tables 1-7 [14]. Abbreviations: PPGL, pheochromocytoma/paraganglioma; SDHx, pathogenic variant in a succinate dehydrogenase gene; FH, pathogenic variant in a fumarate hydratase gene; PBMC, peripheral mononuclear cells.

Patients with a past history or presence of PPGL were enrolled in 3 clinical studies, the multicenter prospective monoamine-producing tumor study (PMT), PROSPHEO (NCT03344016), or through registry and biobanking protocols of the European Network for the Study of Adrenal Tumours (ENSAT). Two separate patient cohorts were investigated (Fig. 1). Cohort A contained patients enrolled under PMT, from whom heparin plasma (n = 38) and 24-hour urine specimens (n = 36) were collected before and/or after the removal of PPGLs (numbers are detailed in Fig. 1). Cohort B consisted of patients enrolled in Dresden through ENSAT and PROSPHEO, from whom EDTA plasma (n = 45) and blood cells (n = 25) were collected before and/or after the removal of PPGLs (numbers are detailed in Fig. 1 and Supplementary Tables S1-S7 [14]) or from asymptomatic *SDHx* PV carriers (n = 4). Only for 5 patients of cohort B were paired samples of before and after surgery available. Blood samples were taken after an overnight fast. Clinical data included sex, age, germline PV, body mass index (BMI), presence of diabetes mellitus, and tumor volume (determined according to at least 2 recorded dimensions [15]). This work was conducted in accordance with the principles of the Declaration of Helsinki. All patients were enrolled into clinical protocols approved by local review boards in Dresden, Munich, Würzburg, Warsaw, and Nijmegen. All patients signed informed consent.

### Genetic Testing

Germline PVs in *SDHA, SDHB, SDHC, SDHD, SDHAF2*, and other genes predisposing to PPGL (*VHL*, *TMEM127*, *MAX*, *NF1*, and *RET*) were identified using next-generation sequencing and/or Sanger sequencing and multiplex ligation-dependent probe amplification or custom array comparative genomic hybridization detecting large-scale deletions. Genetic testing was performed in all patients either at local centers or at the CNIO, Madrid, Spain. Classification of germline variants aligned with current practice guidelines [16]. Patients included in this study had variants in *SDHx* genes classified as pathogenic or likely pathogenic (Supplementary Tables 1-7 [14]). For 2 variants of uncertain significance in *SDHA*, additional functional data, namely, measurements of SFR in tumor tissue from included patients, were available and led to reclassification as likely pathogenic, since SFRs were strongly elevated compared to a previously determined cutoff [17]. For 2 patients of cohort A the specific gene variant was unknown.

### Preparation of Plasma and Urine Samples

EDTA and heparin blood samples (10 mL) were centrifuged at 4200*g* for 15 minutes at 4 °C (Heraeus Multifuge 1S-R). Plasma and 24-hour urine were aliquoted and stored until further analysis at −80 °C.

Samples of 20 µL undiluted plasma and 20 µL of a 1:10 dilution in water were homogenized with 10 µL of internal standard solution [18] and 400 µL of ice-cold methanol to facilitate protein precipitation. After centrifugation (16 000*g*, 5 minutes, 4 °C), clear supernatants were transferred into a 96-well plate and dried using a vacuum-assisted centrifuge (35 °C, 3 hours), followed by reconstitution in 100 µL of initial mobile phase (0.1% formic acid in water/0.1% formic acid in acetonitrile, 99/1, v/v) and sample injection into the liquid chromatography–tandem mass spectrometry (LC-MS/MS) system.

Urine samples were centrifuged (4500*g*, 5 minutes, 4 °C), diluted 1:10 and 1:50 in water, and 20 µL of the dilutions were homogenized with 10 µL of internal standard solution [18] and 400 µL of ice-cold methanol. After centrifugation (16 000*g*, 5 minutes, 4 °C), clear supernatants were dried and reconstituted as described above for plasma samples.

### Preparation of Erythrocytes and Metabolite Extraction

EDTA blood samples were centrifuged at 4200*g* for 15 minutes at 4 °C (Heraeus Multifuge 1S-R) within 4 hours of sample collection and refrigerated storage. Erythrocytes were then collected with care from the very bottom of the tube to avoid contamination with leukocytes. Purity of the erythrocyte preparation was checked visually under a microscope. A volume of 5 µL erythrocytes was mixed with 500 µL of ice-cold methanol and stored at −20 °C for 20 minutes. After centrifugation at 16 000*g* for 5 minutes at 4 °C, the clear supernatant was divided into 2 aliquots (20 µL and 450 µL), transferred to new reaction tubes, and mixed with 10 µL of internal standard solution. Samples were dried in a vacuum centrifuge (Savant Speedvac SPD 210) and stored at −80 °C until further processing. Additionally, 50 µL of erythrocytes were stored in 1 mL freezing medium (PromoCell) at −80 °C for protein isolation.

### Preparation of Peripheral Blood Mononuclear Cells

Lymphoprep™ density gradient medium (Stem Cell Technologies) was aliquoted at 15 mL in a 50-mL tube. EDTA blood was diluted 1:2 with phosphate-buffered saline (PBS, Gibco). Subsequently, the dilution was layered onto the Lymphoprep density gradient medium. The tube was centrifuged at 800*g* for 20 minutes with disabled deceleration (Rotina 380, Hettich centrifuges). PBMCs located on top of the density gradient medium were collected and transferred to a new tube. PBMCs were washed twice with 15 mL PBS and centrifuged at 500*g* for 10 minutes. After the second wash step, cells were resuspended in 5 mL PBS and counted using a Neubauer chamber (C-Chip, NanoEntek). The cell suspension was centrifuged for another 10 minutes at 120*g* with decreased acceleration speed to remove thrombocytes. PBMCs (1 × 10^6^ cells/mL) were lysed in 500 µL of ice-cold methanol, kept at −20 °C for 20 minutes and centrifuged at 16 000*g* for 5 minutes at 4 °C. The clear supernatant was divided in 2 aliquots (20 µL and 450 µL), transferred to new reaction tubes, and mixed with 10 µL of internal standard solution. Samples were dried in a vacuum centrifuge and stored at −80 °C until further processing.

### Protein Isolation From Erythrocytes and Western Blot

#### Preparation of the membrane fraction

Erythrocytes were thawed, immediately transferred to cell culture medium (RPMI 1640, Gibco) containing 10% horse serum, 5% fetal bovine serum, and 1% Glutamax and centrifuged at 4200*g* for 10 minutes. The supernatant was removed and the pellet was washed in cold PBS. Following the protocol by Wilson et al [19], cells were washed twice with iso-osmotic buffer (103 mM disodium phosphate [Na_2_HPO_4_], 155 mM monosodium phosphate [NaH_2_PO_4_], pH 7.4) and afterwards lysed in 10 mL cell lysis buffer (1:20 dilution of iso-osmotic buffer, 1x protease inhibitor cocktail (Sigma Aldrich), 0.5 mM phenylmethylsulfonylfluoride). Cells were centrifuged at 4200*g*, 4 °C for 10 minutes. This lysis step was repeated until the red cell pellet turned completely white, resembling the membrane fraction of erythrocytes.

#### Sample preparation for SDS-PAGE

The membrane fraction was resuspended in 35 µL radioimmunoprecipitation assay buffer (RIPA, Serva) containing 1x protease inhibitor cocktail and chilled on ice for 20 minutes. After centrifugation at 16 000*g*, 5 minutes, 4 °C, supernatant was transferred to a new reaction tube, mixed with 4-fold Laemmli buffer (Bio-Rad) and 5% β-mercaptoethanol and denatured at 100 °C for 5 minutes.

#### Western blot

Proteins were resolved by SDS-PAGE on 10% polyacrylamide gels and transferred to a 0.45 µm polyvinylidene difluoride membrane (Amersham™ Hybond™, Cytiva) by semi-dry electroblotting. Membranes were blocked by incubation with 5% skimmed milk powder in TBS-T (0.05 M Tris, 0.15 M sodium chloride, 0.1 M hydrochloric acid, 1:1000 Polysorbat 20 [Tween20, Serva]) at room temperature for 1 hour, followed by incubation with primary antibody, including anti-SDHB (1:1000, Sigma Aldrich, HPA002868, RRID: AB_1079889) and anti-β-actin (1:1000, Millipore, MAB1501R, RRID: AB_2223041) for 2 hours at room temperature and subsequent incubation overnight. After 3 washing-steps (20 minutes each) in TBS-T, membranes were incubated at room temperature for 1 hour with peroxidase-conjugated secondary antibody goat anti-mouse IgG (1:5000, Santa Cruz, sc-2005, RRID: AB_631736) or goat anti-rabbit IgG (1:5000, Santa Cruz, sc-2004, RRID: AB_631746), diluted in 5% skimmed milk powder in TBS-T. After 3 washing steps (30 minutes each) in TBS-T, proteins were visualized by chemiluminescence using SuperSignal® West Pico and Atto chemiluminescence substrates (Thermo Fisher Scientific) and imaged using the Fusion FX6 EDGE (Vilber Smart Imaging). Densitometry was performed using ImageJ software, protein bands of each sample were analyzed, and ratios were calculated against β-actin.

### Liquid Chromatography–Tandem Mass Spectrometry

Metabolites (succinate, fumarate, malate, citrate, isocitrate, cis-aconitate, α-ketoglutarate, 2-hydroxyglutarate, glutamate, aspartate, glutamine, asparagine, and lactate) were quantified using a previously described LC-MS/MS method [18].

### Nuclear Magnetic Resonance Spectroscopy

Frozen samples were thawed at room temperature for 30 minutes before being mixed with phosphate buffer containing internal reference trimethylsilylpropanoic acid (300 µL each) and urine samples with a phosphate buffer (540 µL to 60 µL) to a volume of 600 µL. The resulting suspension was transferred into nuclear magnetic resonance (NMR) sample tubes and loaded onto the machine. All NMR measurements were run on a Bruker 600 MHz Avance III Neo equipped with a BBI Probe and a Bruker SampleJet robot with a cooling system for sample storage at 4 °C. The plasma samples were measured at 310 K and urine samples at 300 K. A full quantitative calibration was completed before the measurement. All measurements followed the Bruker in vitro diagnostics (IVDr) standard operating procedures and methods [20]. All data were processed in automation using Bruker TopSpin 4.1.1 and ICON NMR. Automatic metabolite reports were obtained using Bruker IVDr B.I.Methods Plasma (B.I.Quant-PS, v2.0.0) and B.I.Methods extended Urine (B.I.Quant-UR E 1.1.0).

### Statistics and Visualization

Data were analyzed and visualized with the software package JMP Pro 17. Normal distribution was tested by Shapiro-Wilk analysis. Nonparametric comparisons of numeric data were performed with the Wilcoxon rank sums test and paired analysis with Wilcoxon signed-rank test. Significance was considered for *P* < .05. Multiple testing correction with a false discovery rate of 0.05 was applied and displayed as adjusted *P* value. Correlations between numerical data were estimated by linear regression. Least squares regression analysis was used to investigate predictive variables (sex, age, diagnosed diabetes mellitus, BMI, tumor presence, and *SDHx* gene) for measured metabolite levels. Diagnostic performance for separating patients with *SDHx* PV from patients without *SDHx* PV was assessed by receiver operating characteristic (ROC) curve analysis. The derived Youden index was used as cutoff for further calculation of diagnostic sensitivity and specificity.

## Results

### Study Population

This study included 2 cohorts of patients, who either had PPGL themselves or had family members with PPGL and hence received genetic testing (Fig. 1, Supplementary Tables 1-7 [14]). In cohort A, all patients had PPGL, 15 individuals had heterozygous germline PVs in *SDHx* genes, 2 patients had somatic *SDHx* aberrations, and 21 patients had no PVs in *SDHx*. Cohort B consisted of 20 individuals with germline PVs in *SDHx*, from whom 4 were asymptomatic, 1 patient with *SDHC* promoter methylation [21], and 24 patients without PVs in *SDHx*, who had a history of PPGL. Distribution of sexes was similar in both cohorts with a roughly equal split between male and female in the *SDHx* PV group, and an overrepresentation of female individuals in the group of patients without *SDHx* PVs (Table 1). Sampling age was lower for patients with *SDHx* PVs in both cohorts. All clinical parameters referred to in this study, including tumor volume, BMI, presence of diabetes mellitus type 2, and erythrocyte characteristics, are summarized in Table 1.

**Table 1.** Participant characteristics

Variable	Cohort A			Cohort B		
	*SDHx* ger. (n = 15)	*SDHx* som. (n = 2)	no *SDHx* (n = 21)	*SDHx* ger. (n = 20)	*SDHx* som. (n = 1)	no *SDHx* (n = 24)
Sex, n (%)						
Female	9 (60)	1 (50)	14 (67)	8 (40)	1	16 (67)
Male	6 (40)	1 (50)	7 (33)	12 (60)	0	8 (33)
Age at sampling, mean (95% CI) [years]*^a^*	38 (30, 46)	(21, 32)	48 (40, 55)	51 (45, 56)	(40)	58 (51, 65)
Mutated gene, n						
SDHA	2	0	—	1	0	—
SDHAF2	0	0	—	1	0	—
SDHB	6	0	—	10	0	—
SDHC	2	1	—	4	1	—
SDHD	5	1	—	4	0	—
FH	—	—	0	—	—	1
VHL	—	—	1	—	—	2
NF1	—	—	2	—	—	0
RET	—	—	3	—	—	2
No germline PV found	—	—	15	—	—	19
History of PPGL, n (%)	15 (100)	2 (100)	21 (100)	16 (80)	1 (100)	24 (100)
Metastatic disease, n (%)	5 (33)	0	1 (5)	3 (15)	0	0
Tumor volume, mean (95% CI) [cm^3^]*^b^*	32 (4, 16)	(2, 26)	108 (16, 200)	—	—	—
BMI, mean (95% CI)	—	—	—	29 (27, 30)	(34)	27 (25, 30)
D. mellitus T2, n (%)	—	—	—	1 (5)	0	3 (13)
Erythrocyte parameters, mean (95% CI)				n = 19		n = 23
Hemoglobin [g/dL]	—	—	—	13.83 (13.02, 14.65)	—	13.28 (12.57, 14.01)
Hematocrit	—	—	—	0.40, (0.39, 0.43)	—	0.39 (0.37, 0.41)
Red blood cell count [*1012/L]	—	—	—	4.60 (4.34, 4.86)	—	4.47 (4.29, 4.64)
MCH [fmol]	—	—	—	1.87 (1.81, 1.93)	—	1.85 (1.79, 1.90)
MCHC [mmol/L]	—	—	—	21.2 (20.7, 21.6)	—	20.9 (20.7, 21.2)
MCV [fl]	—	—	—	88.3 (85.8, 90.7)	—	88.1 (85.7, 90.4)
RDW [%]	—	—	—	13.1 (12.7,13.5)	—	13.3 (12.9, 13.6)

Abbreviations: BMI, body mass index; ger., germline; MCH, mean corpuscular hemoglobin; MCHC, mean corpuscular hemoglobin concentration; MCV, mean corpuscular volume; PPGL, pheochromocytoma/paraganglioma; PV, pathogenic variant; RDW, red cell distribution width; *SDHx*, succinate dehydrogenase gene; som., somatic;

^*a*^Cohort A: sampling age before surgery.

^*b*^Calculations based on measurements of at least 2 dimensions as described elsewhere (15).

### Plasma SFR Best Separates Patients With and Without *SDHx* PV

**Figure 2.**
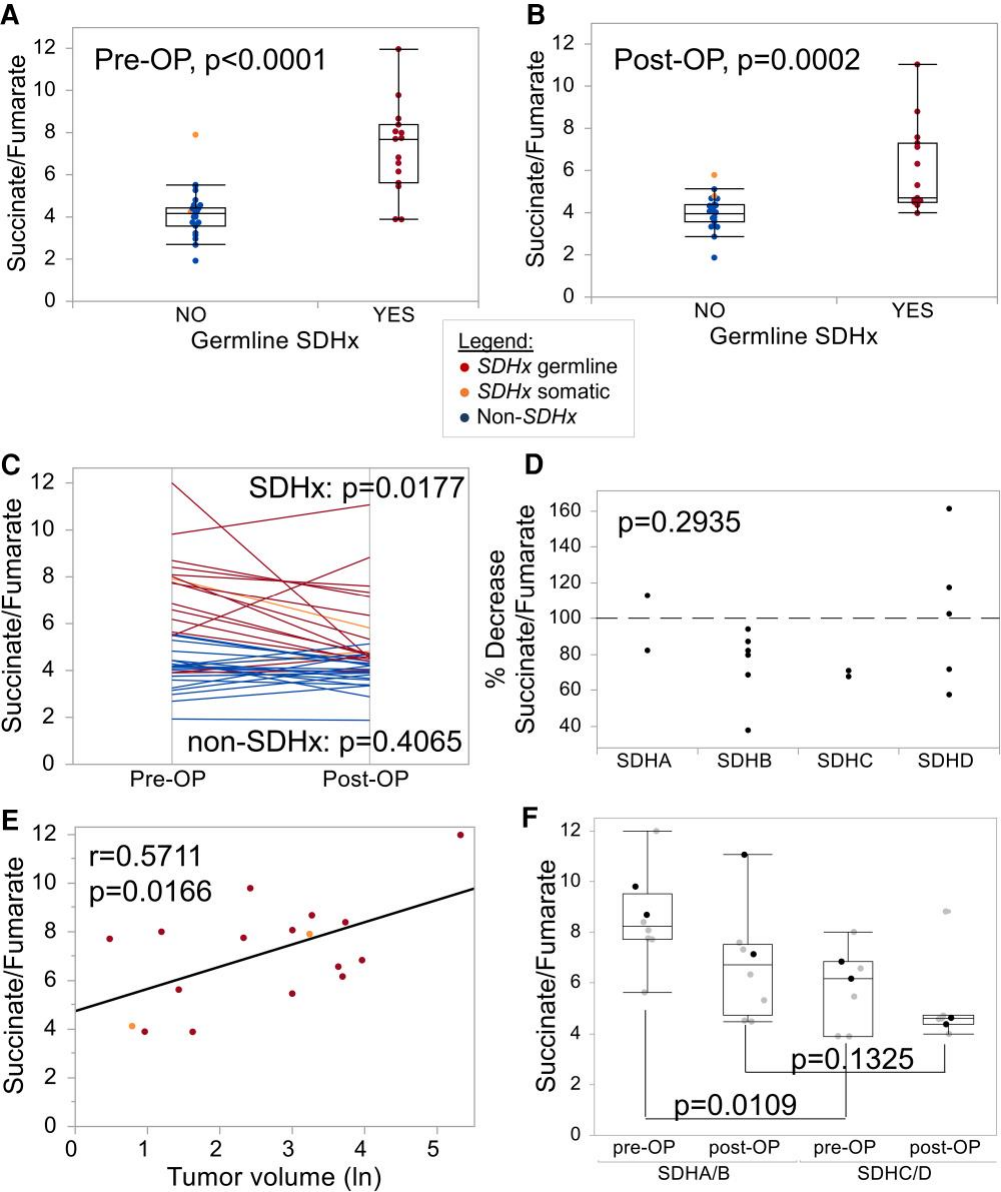
Succinate to fumarate ratio determined in heparin plasma by LC-MS/MS. Plasma was collected from patients with PPGL (cohort A) either preoperatively (Pre-OP, A) or postoperatively (Post-OP, B). C) Paired analysis between pre-OP and post-OP samples; *P* value represents Wilcoxon signed-rank test (1-tailed for SDHx, 2-tailed for non-SDHx). D) Decrease in the succinate to fumarate ratio according to genes in % (Kruskal-Wallis test). E) Correlation between pre-OP succinate to fumarate ratios and tumor volume (natural logarithm of value in cm^3^ displayed). F. Succinate to fumarate ratio according to the affected gene comparing patients with germline *SDHA* (black dots) and *SDHB* (gray dots) PVs to patients with *SDHC* (black dots) and *SDHD* (gray dots) PVs (Kruskal-Wallis test).

Metabolites were measured in heparin plasma (cohort A) using 2 technologies: a LC-MS/MS approach for the assessment of low-molecular organic acids of the Krebs cycle, and NMR spectroscopy, which quantifies a broader spectrum of metabolites. All raw data are presented in Supplementary Tables 1-7 [14]. The SFR determined by LC-MS/MS was significantly different between patients with and without *SDHx* PV, both in plasma collected preoperatively and postoperatively (Fig. 2A and 2B). The SFR was generally lower after tumor removal in 11 of 15 patients with germline *SDHx* PV and in 1 of 2 patients with somatic *SDHx* aberration (Fig. 2C). Metastatic disease was not the reason for persistently high or higher than baseline SFRs. A failed drop in SFR occurred more often in patients with *SDHD* PV, but not exclusively (Fig. 2D). Furthermore, decreased SFR after surgery occurred in 8 of 9 female patients, but only in 3 of 6 male patients. Preoperative SFR correlated significantly with tumor volume, indicating that at least some of the plasma metabolites originate from tumor cells (Fig. 2E). Significantly higher SFRs were measured preoperatively in plasma from patients with *SDHA* or *SDHB* PV compared to patients with *SDHC* or *SDHD* PV (Fig. 2F). Succinate and metabolite ratios containing succinate were the only significant differences between patients with and without *SDHx* PV preoperatively and postoperatively (Table 2).

**Table 2.** Diagnostic power of succinate and ratios to differentiate between patients with and without SDHx PV in the presence (pre-OP) and absence (post-OP) of PPGL

Tumor status	Matrix	Parameter*^a^*	*P* value*^b^*	AUC of ROC curve*^c^*
Pre-OP	Heparin plasma (n = 38)	Succinate	<.0001	0.907 (0.815, 0.999)
		Succinate/Fumarate	<.0001	0.887 (0.786, 0.988)
		Succinate/Malate	<.0001	0.907 (0.815, 0.999)
		Succinate/Citrate	<.0001	0.910 (0.819, 1001)
		Succinate/Asparagine	<.0001	0.849 (0.735, 0.963)
	EDTA plasma (n = 18)	Succinate	.0050	0.900 (0.817, 0.983)
		Succinate/Fumarate	<.0001	0.900 (0.817, 0.983)
		Succinate/Malate	.0255	0.775 (0.659, 0.891)
		Succinate/Citrate	.0004	0.938 (0.871, 1.005)
		Succinate/Asparagine	.0012	0.888 (0.801, 0.975)
Post-OP + asymptomatic carriers	Heparin plasma (n = 38)	Succinate	.0111	0.699 (0.553, 0.845)
		Succinate/Fumarate	<.0001	0.867 (0.759, 0.975)
		Succinate/Malate	.0001	0.852 (0.739, 0.965)
		Succinate/Citrate	<.0001	0.849 (0.735, 0.963)
		Succinate/Asparagine	.0013	0.765 (0.630, 0.900)
	EDTA plasma (n = 31)	Succinate	.0002	0.873 (0.781, 0.969)
		Succinate/Fumarate	<.0001	0.947 (0.885, 1.009)
		Succinate/Malate	<.0001	0.956 (0.899, 1.013)
		Succinate/Citrate	.0649	0.794 (0.682, 0.906)
		Succinate/Asparagine	.3685	0.798 (0.687, 0.909)

^*a*^Logistic regression analysis.

^*b*^Effect likelihood ratio test.

^*c*^Area under the receiver operating characteristic curve (95% CI).

NMR spectroscopy was unable to detect succinate or fumarate in plasma. In postoperative samples, NMR spectroscopy identified glutamine as the only metabolite to be significantly different between patients with and without *SDHx* PV (adjusted *P* = .026). Glutamine separated these groups with an area under the ROC curve (AUC) of 0.849.

### Diagnostic Performance of SFR in Plasma

**Figure 3.**
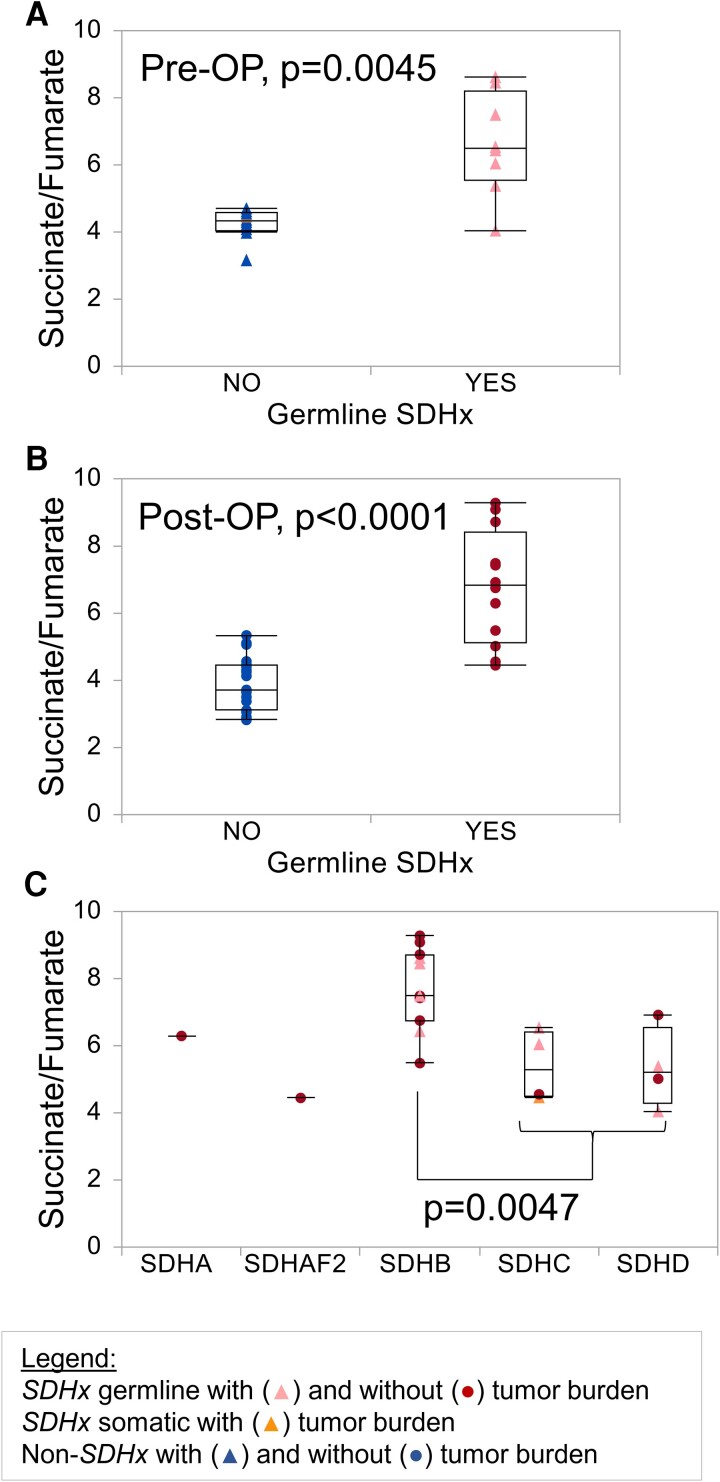
Succinate to fumarate ratio determined in EDTA plasma by LC-MS/MS. Plasma was collected from patients of cohort B either preoperatively (Pre-OP, n = 18, A) or in patients without tumor burden (Post-OP, n = 31, removal of a high succinate outlier in the group of patients with *SDHx* PV, B). Tumor burden refers to the presence of primary tumors or metastases and was determined by anatomical and/or functional imaging. C) Succinate-fumarate-ratios are displayed according to the affected gene. Triangles depict patients with a PPGL, circles show patients without tumor burden. Patients with *SDHx* germline PV, *SDHx* somatic PV, and without *SDHx* PV are shown in red, orange, and blue, respectively.

Analogous to investigations in heparin plasma, metabolites were measured in EDTA plasma (cohort B) by LC-MS/MS. The SFR separated patients with and without *SDHx* PV preoperatively and postoperatively with high statistical significance (Fig. 3A and 3B). Patients with *SDHB* PV showed the highest SFRs (Fig. 3C). Type of gene was the only significantly associated variable by regression analysis among the parameters, tumor presence, sex, age, diagnosed diabetes mellitus, and BMI. In both matrixes the SFR showed high diagnostic performance with AUCs of 0.867 to 0.947 (Table 2). Other possible ratios are succinate/citrate and succinate/malate with AUCs of 0.794 to 0.938 and 0.775 to 0.956, respectively. Succinate alone had AUCs of 0.699 to 0.907. Since our cohorts only included one patient with germline *FH* PV, an analysis of metabolic parameters for the identification of such individuals is not possible; however, asparagine, asparagine/2-hydroxyglutarate, fumarate/2-hydroxyglutarate, malate/2-hydroxyglutarate, and glutamine/2-hydroxyglutarate are possible markers that should be investigated in a larger cohort (Table 3).

**Table 3.** Potential parameters for identification of FH PV carriers in EDTA plasma

Metabolite/Ratio	Patient with FH PV (n = 1)	Patients without FH-PV*^a^* (n = 49)
Asparagine (ng/mL)	7535	5142.8 (935.0-7011.2)
Asparagine/2HG	74.8	24.6 (6.1-58.2)
Fumarate/2HG	1.4	0.6 (0.2-1.1)
Malate/2HG	5.6	2.8 (1.0-5.0)
Glutamine/2HG	1011.5	406.4 (93.9-899.4)

Abbreviations: 2HG, 2-hydroxyglutarate; FH, fumarate hydratase; PV, pathogenic variant.

^*a*^Median and range in parentheses.

### Urinary Metabolites Do Not Discriminate Between Patients With and Without *SDHx* PV

Given that urinary succinate levels were more than 10 times higher than those in plasma (median 62 [range, 11-347] mmol/L vs 4 [2-52] mmol/L), NMR spectroscopy also detected this metabolite. Levels of succinate and the SFR were in a similar range in patients with and without *SDHx* PV. No significant differences between patients with and without *SDHx* PV were detected in urine.

### Erythrocytes but Not PBMCs Differ in Their Metabolism Between Patients With and Without *SDHx* PV

**Figure 4.**
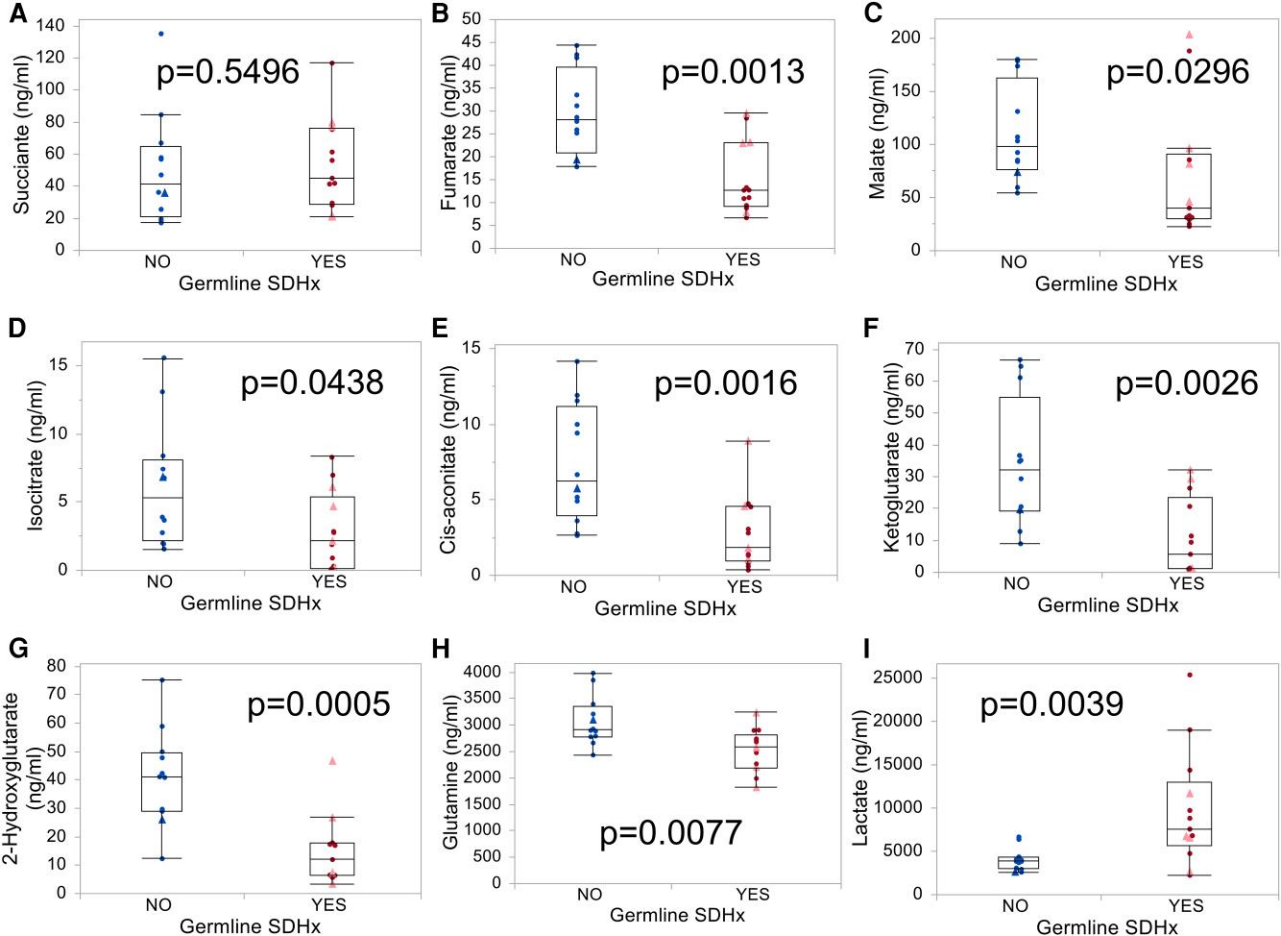
Krebs cycle metabolites in erythrocytes from patients with and without *SDHx* PVs. A-I) Erythrocytes were collected from patients (cohort B) in whom PPGL was present (triangles, light red) or absent (circles, dark red). Patients with *SDHx* germline PV and without *SDHx* PV are shown in red and blue, respectively.

**Figure 5.**
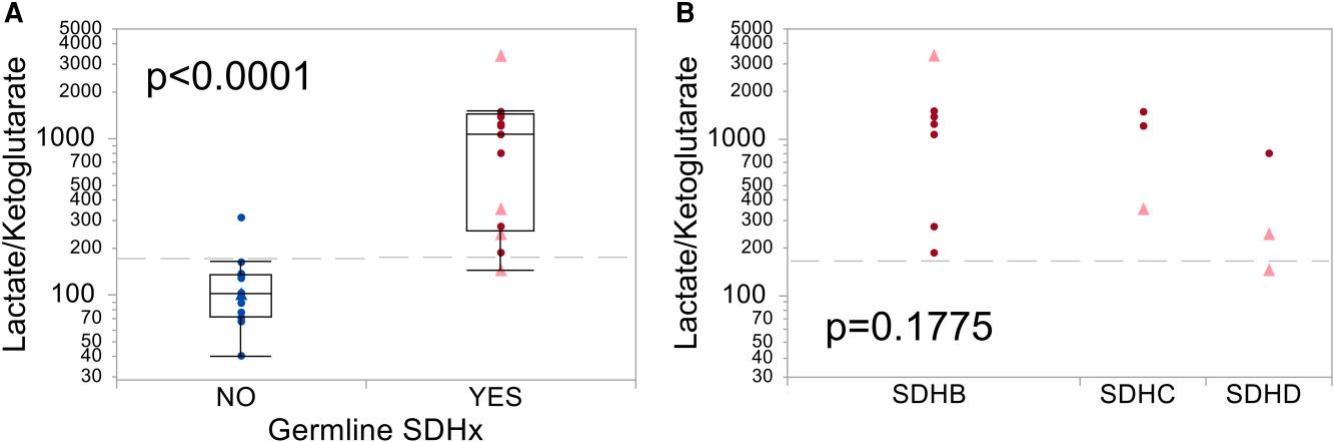
The lactate/α-ketoglutarate-ratio from erythrocytes separates patients with *SDHx* PVs. A) Erythrocytes were collected from patients (cohort B) in whom PPGL was present (triangles, light red) or absent (circles, dark red). Patients with *SDHx* germline PV and without *SDHx* PV are shown in red and blue, respectively. Gray dashed lines indicate cutoff according to Youden index (186). As ratios of lactate/ketoglutarate and lactate/2-hydroxyglutarate show similar results, only the first one is displayed here. B) Lactate/α-ketoglutarate does not significantly differ between patients with different *SDHx* genes. Since the lactate/2-hydroxyglutarate ratio shows exactly the same results as lactate/α-ketoglutarate, only the latter is displayed in this figure.

**Figure 6.**
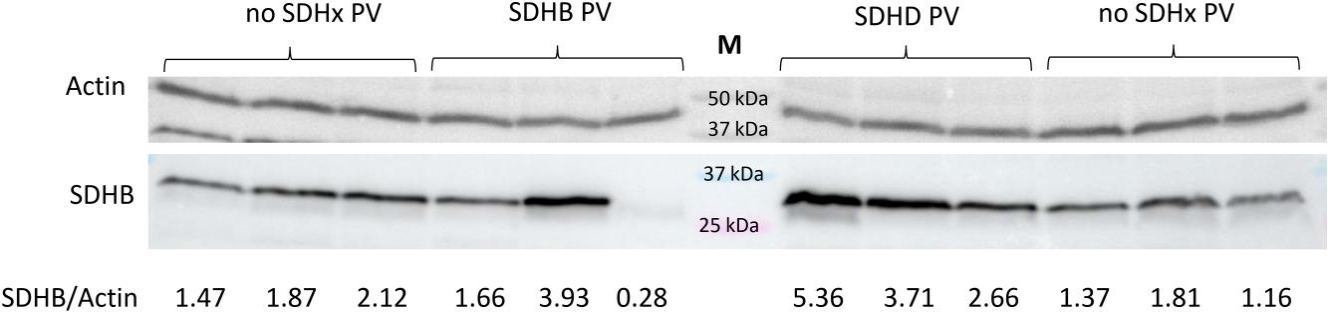
SDHB protein levels in erythrocytes. Membrane fractions of erythrocytes were collected from carriers of *SDHx* PV and controls (cohort B), isolated and analyzed for protein levels by Western blot. One lane represents results for one patient. Full blots and additional patients are available in the supplement (Supplementary Fig. S1 and S2 [14]). Densitometry calculations are provided on the bottom. M—Marker.

Succinate or the SFR did not show differences between patients with and without *SDHx* PV in PBMCs (data not shown) or erythrocytes (Fig. 4A). No significant metabolic alterations were measured in PBMCs, whereas strong differences were detected in erythrocytes of patients with compared to those without germline *SDHx* PV (Fig. 4B-4I). Of 13 quantified metabolites, 8 were significantly different between groups, including fumarate, malate, isocitrate, cis-aconitate, α-ketoglutarate, 2-hydroxyglutarate, lactate, and glutamine. Lactate/α-ketoglutarate and lactate/2-hydroxyglutarate showed the best diagnostic separation of patients with and without *SDHx* PV with an AUC of 0.968 (Table 4, Fig. 5A). Interestingly, these erythrocyte parameters appear to be to a lesser extent influenced by gene type (Fig. 5B). Combination of SFR in EDTA plasma and lactate/α-ketoglutarate in erythrocytes yielded a marginal improvement of specificity over either parameter alone, reaching a specificity of 100% and sensitivity of 92% (Table 5). Analysis of patient blood counts did not yield any significant differences in erythrocyte parameters between carriers with *SDHx* PVs and other patients (Table 1). Furthermore, we did not detect any consistent differences in SDHB protein levels in membrane fractions of erythrocytes between individuals with and without *SDHx* PV (Fig. 6).

**Table 4.** Most significantly different metabolite ratios in erythrocytes from patients with vs without SDHx PVs

Parameter*^a^*	*P* value*^b^*	AUC of ROC curve*^c^*
Lactate/Ketoglutarate	<.0001	0.968 (0.899, 1.037)
Lactate/2HG	<.0001	0.968 (0.899, 1.037)
Lactate/Cis-aconitate	<.0001	0.962 (0.887, 1.037)
Lactate/Fumarate	<.0001	0.936 (0.840, 1.032)
2HG/Asparagine	<.0001	0.923 (0.818, 1.028)
Asparagine/Fumarate	<.0001	0.923 (0.818, 1.028)
Lactate/Malate	.0001	0.910 (0.798, 1022)
2HG/Glutamine	.0002	0.910 (0.798, 1022)
2HG/Succinate	.0002	0.910 (0.798, 1022)
Fumarate	.0002	0.878 (0.750, 1006)
Lactate	.0018	0.840 (0.696, 0.984)

Abbreviations: 2HG, 2-hydroxyglutarate; AUC, area under the curve, PV, pathogenic variant; ROC, receiver operator characteristic.

^*a*^Only most significant metabolite ratios with AUC > 0.9 are listed.

^*b*^Likelihood ratio test.

^*c*^95% CIs in parentheses.

**Table 5.** Diagnostic performance of metabolite ratios in plasma and erythrocytes to identify patients with SDHx PVs

Parameter	AUC of ROC curve	Specificity	Sensitivity
EDTA-Succinate/Fumarate	0.929	92% (11/12)	92% (12/13)
Erythrocyte-Lactate/Ketoglutarate	0.968	92% (11/12)	92% (12/13)
Combined	0.955	100% (12/12)	92% (12/13)

Specificity/sensitivity were calculated based on the Youden index of ROC curves.

Abbreviations: AUC, area under the curve, PV, pathogenic variant; ROC, receiver operator characteristic.

## Discussion

This study shows for the first time that not only plasma from patients with and without *SDHx* PVs is distinct, but also erythrocytes have different features. PBMCs, on the other hand, were largely unchanged. Our findings open up new diagnostic options for the identification of carriers with *SDHx* PVs and could aid in classification of VUS in *SDHx* genes. ClinVar currently has more than 3000 entries for VUS in *SDHx* genes (https://www.ncbi.nlm.nih.gov/clinvar, visited June 25, 2024). Relatives of patients with *SDHx*-related PPGL in whom a VUS was identified are not eligible for genetic testing and will not be screened regularly for tumors. In these cases, a blood-based test could provide more clarity. Additionally, the proposed assay would be useful in settings where genetic testing is not available, or delayed, or patients refuse genetic analysis. Due to higher sensitivity of LC-MS/MS compared to NMR, LC-MS/MS is the preferred technique for measurements of carbon metabolites, especially in plasma, where succinate levels are 10-fold lower compared to urine.

Our investigation extends results from 2 previous studies that showed succinate or the SFR in serum is elevated in patients with *SDHB* PVs, both in the presence of PPGL and in asymptomatic patients [12, 13]. Patients with PVs in other subunits of succinate dehydrogenase did not show a significant increase in those studies. Our results confirm that individuals with *SDHB* but also *SDHA* PVs have the highest plasma succinate levels. This influence was even stronger than the presence of PPGL. Whether plasma SFR will be a useful biomarker for tumor development in carriers of *SDHx* PVs and whether this assay could reduce the need for expensive whole-body imaging will have to be investigated in the future.

Other previously proposed parameters influencing succinate levels, such as obesity and diabetes mellitus type 2, were not significant factors in our study [22]. The source of succinate in tumor-free *SDHx* PV carriers is unknown but might originate from metabolically active and energy-demanding cells, such as liver or muscle. Recently, succinate was described as a paracrine regulator of muscle remodeling after exercise [23], raising the question whether carriers of *SDHx* PVs have altered responses to exercise. Lower levels of plasma succinate in individuals with *SDHC* and *SDHD* PVs could indicate that a lesser expression of the membrane bound subunits *SDHC* and *SDHD* might be more easily compensated than reduction in subunits facilitating catalytic activity.

Correlation of plasma SFR with tumor volume supports a previous study, in which serum succinate was shown to function as a biomarker for tumor recurrence and tumor burden in 3 patients with metastatic *SDHB*-related PPGL [12]. SFR could be easily measured in the same sample and with the same instrumentation used for plasma metanephrines [24] and would provide another parameter for evaluating treatment success or recurrence in patients with *SDHx*-related PPGL. Our analysis indicates that, beyond SFR, also other ratios with succinate could be utilized, specifically succinate/citrate and succinate/malate, which showed similar results.

Despite succinate elevations in plasma, urinary succinate or SFR were similar between patients with and without *SDHx* PVs. Urinary sources of succinate might originate from cell populations less responsive to SDH loss and might only occur in pathological settings. To this end, increases in urinary succinate were linked previously to reduced mitochondrial metabolism and more severe kidney disease [25].

A previous study reported on increased succinate and lactate in neutrophils of patients with *SDHB* PVs [26]. PBMCs, on the other hand, did not show any difference between patients with and without *SDHx* PVs in our study. As succinate drives proinflammatory responses in macrophages [27], there may be regulatory safety nets to prevent succinate elevations at baseline. Granulocytes were not investigated in the present study.

Although mature erythrocytes are devoid of mitochondria, SDHB is present in their membrane fraction [19]. Furthermore, it was demonstrated that high altitude affects levels of carboxylic acids of the central carbon metabolism in erythrocytes, including α-ketoglutarate, succinate, and others [28]. Our study found that erythrocytes from individuals with *SDHx* PV have lower levels of fumarate, malate, isocitrate, cis-aconitate, α-ketoglutarate, 2-hydroxyglutarate, and glutamine, but higher levels of lactate compared to controls. Based on the increased lactate to α-ketoglutarate ratio, we hypothesize that glycolytic flux may be increased in erythrocytes from patients with *SDHx* PV by favoring glycolysis over the pentose phosphate pathway, which is responsible for maintaining antioxidant capacity [29]. Mice with heterozygous deletions of different *SDHx* genes show an increased healthy life span under hypobaric oxygen with reduced red cell distribution width [30]. This may be facilitated by an improved glycolytic flux that most likely results in higher energy levels under low oxygen conditions in erythrocytes with *SDHx* loss compared to controls. A recent observation of an inheritance ratio of 0.6 for *SDHB* PVs suggests that heterozygous *SDHB* loss has developmental advantages [31]. As different regions of the embryo are exposed to hypoxia during development [32], it could be conceivable that more hypoxia-resistant cells may be selected for.

The present study is a proof-of-concept for the combination of plasma and erythrocyte parameters, and it suffers from a number of limitations. Foremost, the relatively small number of patients included in our analysis complicates investigations toward factors with more subtle influence on plasma SFR levels. Furthermore, the lack of a large comparison group of healthy individuals hinders the establishment of reference intervals for diagnostic use. The present study only analyzed blood samples from a maximum of 2 time points and did not follow individual patients through their course of disease. Hence, we cannot comment on how plasma SFR behaves in longitudinal follow-up, for which imaging information as comparison would be crucial. The latter were not included in the present analysis; instead, tumor burden was assessed based on volume calculations [15]. Systematic investigations on what factors influence physiological levels of succinate and fumarate were not part of this study. To account for food-related effects, samples were taken in line with pre-analytics for metanephrines after an overnight fast. As exercise and BMI were previously implicated in plasma succinate variations [22, 23], individual levels of succinate may be associated with muscle mass and fat depots.

This study shows for the first time that not only liquid parts of a blood sample carry valuable information about potentially pathogenic variants in one of the *SDHx* genes, but also erythrocytes have diagnostic power. As only a small number of patients (n = 25) were investigated for changes in erythrocyte metabolism, it will be important to follow up with a prospective study, as the described method could be valuable for early identification of PV carriers in all *SDHx* genes. Additionally, our results highlight that heterozygous PVs in *SDHx* genes lead to physiological changes due to loss of one *SDHx* allele. Further insights into these physiological adaptations might uncover whether carriers are at a higher or possibly lower risk for developing other more common diseases.

## Data Availability

Datasets of metabolic measurements were deposited online at Zenodo: https://zenodo.org/records/12742032.

## Abbreviations

AUC: area under receiver operator characteristic curve
BMI: body mass index
LC-MS/MS: liquid chromatography–tandem mass spectrometry
NMR: nuclear magnetic resonance
PBMC: peripheral blood mononuclear cell
PBS: phosphate-buffered saline
PPGL: pheochromocytoma/paraganglioma
PV: pathogenic variant
ROC: receiver operating characteristic
SDH: succinate dehydrogenase
SFR: succinate to fumarate ratio
VUS: variant of uncertain significance

## References

[1] MacFarlane J , Seong KC, Bisambar C, et al A review of the tumour spectrum of germline succinate dehydrogenase gene mutations: beyond phaeochromocytoma and paraganglioma. Clin Endocrinol. 2020;93(5):528‐538. 10.1111/cen.14289 32686200

[2] Amar L , Pacak K, Steichen O, et al International consensus on initial screening and follow-up of asymptomatic SDHx mutation carriers. Nat Rev Endocrinol. 2021;17(7):435‐444. 34021277 10.1038/s41574-021-00492-3 PMC8205850

[3] Buffet A , Ben Aim L, Leboulleux S, et al Positive impact of genetic test on the management and outcome of patients with paraganglioma and/or pheochromocytoma. J Clin Endocrinol Metab. 2019;104(4):1109‐1118. 30698717 10.1210/jc.2018-02411

[4] Davidoff DF , Benn DE, Field M, et al Surveillance improves outcomes for carriers of sdhb pathogenic variants: a multicenter study. J Clin Endocrinol Metab. 2022;107(5):e1907‐e1916. 35037935 10.1210/clinem/dgac019 PMC9016424

[5] Benn DE , Zhu Y, Andrews KA, et al Bayesian approach to determining penetrance of pathogenic SDH variants. J Med Genet. 2018;55(11):729‐734. 30201732 10.1136/jmedgenet-2018-105427 PMC6252366

[6] Rijken JA , Niemeijer ND, Jonker MA, et al The penetrance of paraganglioma and pheochromocytoma in SDHB germline mutation carriers. Clin Genet. 2018;93(1):60‐66. 28503760 10.1111/cge.13055

[7] Maniam P , Zhou K, Lonergan M, Berg JN, Goudie DR, Newey PJ. Pathogenicity and penetrance of germline SDHA variants in pheochromocytoma and paraganglioma (PPGL). J Endocr Soc. 2018;2(7):806‐816. 29978154 10.1210/js.2018-00120 PMC6030830

[8] Andrews KA , Ascher DB, Pires DEV, et al Tumour risks and genotype-phenotype correlations associated with germline variants in succinate dehydrogenase subunit genes SDHB, SDHC and SDHD. J Med Genet. 2018;55(6):384‐394. 29386252 10.1136/jmedgenet-2017-105127 PMC5992372

[9] Richter S , Garrett TJ, Bechmann N, Clifton-Bligh RJ, Ghayee HK. Metabolomics in paraganglioma: applications and perspectives from genetics to therapy. Endocr Relat Cancer. 2023;30(6):e220376. 36897220 10.1530/ERC-22-0376 PMC10228374

[10] Richter S , Gieldon L, Pang Y, et al Metabolome-guided genomics to identify pathogenic variants in isocitrate dehydrogenase, fumarate hydratase, and succinate dehydrogenase genes in pheochromocytoma and paraganglioma. Genet Med. 2019;21(3):705‐717. 30050099 10.1038/s41436-018-0106-5 PMC6353556

[11] Kim E , Wright MJ, Sioson L, et al Utility of the succinate: fumarate ratio for assessing SDH dysfunction in different tumor types. Mol Genet Metab Rep. 2017;10:45‐49. 28070496 10.1016/j.ymgmr.2016.12.006 PMC5219629

[12] Lamy C , Tissot H, Faron M, et al Succinate: a serum biomarker of SDHB-mutated paragangliomas and pheochromocytomas. J Clin Endocrinol Metab. 2022;107(10):2801‐2810. 35948272 10.1210/clinem/dgac474

[13] Bancel LP , Masso V, Dessein AF, et al Serum succinate/fumarate ratio in patients with paraganglioma/pheochromocytoma attending an endocrine oncogenetic unit. J Clin Endocrinol Metab. 2023;108(9):2343‐2352. 36848172 10.1210/clinem/dgad109

[14] Gebhardt M , Kunath C, Fröbel D, et al Identification of succinate dehydrogenase gene variant carriers by blood biomarkers (Supplement). 2024. Doi:10.5281/zenodo.12742032 PMC11323779 39145115

[15] Eisenhofer G , Deutschbein T, Constantinescu G, et al Plasma metanephrines and prospective prediction of tumor location, size and mutation type in patients with pheochromocytoma and paraganglioma. Clin Chem Lab Med. 2020;59(2):353‐363. 33001846 10.1515/cclm-2020-0904

[16] Richards S , Aziz N, Bale S, et al Standards and guidelines for the interpretation of sequence variants: a joint consensus recommendation of the American College of Medical Genetics and Genomics and the Association for Molecular Pathology. Genet Med. 2015;17(5):405‐424. 25741868 10.1038/gim.2015.30 PMC4544753

[17] Richter S , Peitzsch M, Rapizzi E, et al Krebs cycle metabolite profiling for identification and stratification of pheochromocytomas/paragangliomas due to succinate dehydrogenase deficiency. J Clin Endocrinol Metab. 2014;99(10):3903‐3911. 25014000 10.1210/jc.2014-2151 PMC4184070

[18] Wallace PW , Conrad C, Bruckmann S, et al Metabolomics, machine learning and immunohistochemistry to predict succinate dehydrogenase mutational status in phaeochromocytomas and paragangliomas. J Pathol. 2020;251(4):378‐387. 32462735 10.1002/path.5472 PMC7548960

[19] Wilson MC , Trakarnsanga K, Heesom KJ, et al Comparison of the proteome of adult and cord erythroid cells, and changes in the proteome following reticulocyte maturation. Mol Cell Proteomics. 2016;15(6):1938‐1946. 27006477 10.1074/mcp.M115.057315 PMC5083095

[20] Dona AC , Jimenez B, Schafer H, et al Precision high-throughput proton NMR spectroscopy of human urine, serum, and plasma for large-scale metabolic phenotyping. Anal Chem. 2014;86(19):9887‐9894. 25180432 10.1021/ac5025039

[21] Richter S , Klink B, Nacke B, et al Epigenetic mutation of the succinate dehydrogenase C promoter in a patient with two paragangliomas. J Clin Endocrinol Metab. 2016;101(2):359‐363. 26652933 10.1210/jc.2015-3856

[22] Serena C , Ceperuelo-Mallafre V, Keiran N, et al Elevated circulating levels of succinate in human obesity are linked to specific gut microbiota. ISME J. 2018;12(7):1642‐1657. 29434314 10.1038/s41396-018-0068-2 PMC6018807

[23] Reddy A , Bozi LHM, Yaghi OK, et al pH-Gated succinate secretion regulates muscle remodeling in response to exercise. Cell. 2020;183(1):62‐75.e17. 32946811 10.1016/j.cell.2020.08.039 PMC7778787

[24] Peitzsch M , Prejbisz A, Kroiss M, et al Analysis of plasma 3-methoxytyramine, normetanephrine and metanephrine by ultraperformance liquid chromatography-tandem mass spectrometry: utility for diagnosis of dopamine-producing metastatic phaeochromocytoma. Ann Clin Biochem. 2013;50(Pt 2):147‐155. 23512172 10.1258/acb.2012.012112

[25] Hallows KR , Abebe KZ, Li H, et al Association of longitudinal urinary metabolic biomarkers with ADPKD severity and response to metformin in TAME-PKD clinical trial participants. Kidney Int Rep. 2023;8(3):467‐477. 36938071 10.1016/j.ekir.2022.11.019 PMC10014337

[26] Jones R , McDonald KE, Willson JA, et al Mutations in succinate dehydrogenase B (SDHB) enhance neutrophil survival independent of HIF-1alpha expression. Blood. 2016;127(21):2641‐2644. 27006389 10.1182/blood-2016-02-696922 PMC4965837

[27] Mills EL , Kelly B, Logan A, et al Succinate dehydrogenase supports metabolic repurposing of mitochondria to drive inflammatory macrophages. Cell. 2016;167(2):457‐70.e13. 27667687 10.1016/j.cell.2016.08.064 PMC5863951

[28] Nemkov T , Sun K, Reisz JA, et al Metabolism of citrate and other carboxylic acids in erythrocytes as a function of oxygen saturation and refrigerated storage. Front Med (Lausanne). 2017;4:175. 29090212 10.3389/fmed.2017.00175 PMC5650965

[29] D'Alessandro A , Xia Y. Erythrocyte adaptive metabolic reprogramming under physiological and pathological hypoxia. Curr Opin Hematol. 2020;27(3):155‐162. 32141895 10.1097/MOH.0000000000000574 PMC8900923

[30] Baysal BE , Alahmari AA, Rodrick TC, et al Succinate dehydrogenase inversely regulates red cell distribution width and healthy life span in chronically hypoxic mice. JCI Insight. 2022;7(17):e158737. 35881479 10.1172/jci.insight.158737 PMC9536274

[31] Davidoff DF , Lim ES, Benn DE, et al Distortion in transmission of pathogenic SDHB- and SDHD-mutated alleles from parent to offspring. Endocr Relat cancer. 2023;30(5):e220233. 36786389 10.1530/ERC-22-0233

[32] Dunwoodie SL . The role of hypoxia in development of the Mammalian embryo. Dev Cell. 2009;17(6):755‐773. 20059947 10.1016/j.devcel.2009.11.008

